# Evaluating incident learning systems and safety culture in two radiation oncology departments

**DOI:** 10.1002/jmrs.563

**Published:** 2021-12-09

**Authors:** Laura Adamson, Rachael Beldham‐Collins, Jonathan Sykes, David Thwaites

**Affiliations:** ^1^ Department of Radiation Oncology Crown Princess Mary Cancer Centre Sydney New South Wales Australia; ^2^ Department of Radiation Oncology Blacktown Cancer & Haematology Centre Sydney New South Wales Australia; ^3^ School of Physics, Institute of Medical Physics University of Sydney Sydney New South Wales Australia; ^4^ Department of Radiation Oncology Nepean Hospital Cancer Care Centre Sydney New South Wales Australia

**Keywords:** Incident learning, incident reporting, quality and safety, radiation oncology, radiation therapy, safety culture

## Abstract

**Introduction:**

Radiation oncology patient pathways are complex. This complexity creates risk and potential for error to occur. Comprehensive safety and quality management programmes have been developed alongside the use of incident learning systems (ILSs) to mitigate risks and errors reaching patients. Robust ILSs rely on the safety culture (SC) within a department. The aim of this study was to assess perceptions and understanding of SC and ILSs in two closely linked radiation oncology departments and to use the results to consider possible quality improvement (QI) of department ILSs and SC.

**Methods:**

A survey to assess perceptions of SC and the currently used ILSs was distributed to radiation oncologists, radiation therapists and radiation oncology medical physicists in the two departments. The responses of 95 staff were evaluated (63% of staff). The findings were used to determine any areas for improvement in SC and local ILSs.

**Results:**

Differences were shown between the professional cohorts. Barriers to current ILS use were indicated by 67% of respondents. Positive SC was shown in each area assessed: 69% indicated the departments practised a no‐blame culture. Barriers identified in one department prompted a QI project to develop a new reporting system and process, improve departmental learning and modify the overall ILS.

**Conclusion:**

An understanding of SC and attitudes to ILSs has been established and used to improve ILS reporting, feedback on incidents, departmental learning and the QA program. This can be used for future comparisons as the systems develop.

## Graphical abstract

The aims were to assess attitudes to and understanding of safety culture (SC) and incident learning systems (ILSs) in two closely‐linked radiation oncology departments using a customised survey. Sixty‐three per cent of staff participated. Barriers identified in one department prompted a quality improvement project to develop a new radiation oncology‐specific reporting system, with a focus on improving departmental learning and modifying the overall ILS.

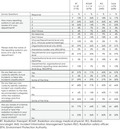

## Introduction

Approximately 50% of Australian cancer patients receive radiation therapy.^1^ The pathway from decision‐to‐treat to completion of a treatment course is highly complex, increasingly so due to continuously developing advanced technologies and techniques. There are many inter‐linking clinical and technical process steps for creating individualised treatment plans and delivering treatment over multiple fractions. Radiation oncology involves a multidisciplinary team (MDT) of radiation oncologists (ROs), radiation oncology medical physicists (ROMPs) and radiation therapists (RTs), including support from oncology nurses and allied health professionals. The MDT work together for high‐quality treatment and patient care, while also minimising risk. However, each activity/step, or interface between steps or information transfer point, has potential for error. Therefore, radiation oncology is subject to detailed quality management and control. Nevertheless, errors may occur.

Errors and near misses are termed ‘incidents’, that is unintentional events or unwanted changes from the normal intended process, which potentially can cause an adverse event.^2^ Further categorisation designates actual incidents or near‐miss incidents.^3^ Actual incidents in radiation oncology are primarily errors where dose delivered to a patient deviates from the prescribed dose or plan, with or without a clinically measurable effect. A near‐miss incident is caught before any incorrect dose is delivered. Near misses can be identified during the pre‐treatment preparation/planning phases or at quality assurance (QA) checkpoints and rectified before a patient treatment begins. They can also be identified while the patient is on the treatment couch, immediately before each treatment delivery, by final QA checks or image guidance procedures. The proportion of treatments where an actual incident has occurred is considered relatively low within radiation oncology. For incidents where deviations are large enough to trigger mandatory reporting into national reporting systems, rates have been estimated at 0.2% per course and for those having clinically significant consequences, estimates are one or two orders of magnitude lower.^4^, ^5^, ^6^ Nevertheless, given the potential consequences of actual incidents, radiation oncology facilities deploy comprehensive QA programs and Incident Learning Systems (ILSs), alongside promoting a positive safety culture (SC).

The Australian and New Zealand bi‐national radiation oncology practice standards (ROPS) recommend robust QA and incident management as requirements to mitigate risk.^7^ Internationally, radiation oncology departments have reported various local ILSs to support this.^5^, ^8^, ^9^, ^10^, ^11^, ^12^ For robust incident evaluation, an ILS should include incident reporting, investigations (e.g. root cause analysis), data tracking, visualisation and practical feedback.^13^ It should also guide quality improvement (QI) and QA practices to guard against similar errors recurring. ILSs that analyse reported data to identify QI areas provide a more proactive and effective response to incident management than simple reactive changes to individual isolated reported incidents.^8^ Departmental ILSs that meet the reporting categories and needs of radiation oncology can impact on reducing error rates and provide appropriate data analysis and practical QI guidance.^6^ ILSs specifically designed for radiation oncology, coupled with continuous assessment and improvement, have reduced occurrence of significant incidents, provided QI insight and facilitated proactive approaches to quality and safety management.^9^, ^10^, ^14^

ILS success relies on the department’s SC. Many studies have shown negativity and frustration from frontline staff regarding incident reporting and learning, more than from management staff.^6^, ^13^, ^15^, ^16^ Negative responses have focussed more on unsatisfactory approaches to investigations, corrective actions and learning from incident reports rather than completing report forms.^16^ Staff show further frustration when ILSs are challenging or time‐consuming to use.^13^ Departmental SC can affect attitudes to an ILS and its utilisation and feedback to staff.

As part of ongoing quality assessment and improvement, staff in the closely linked radiation oncology departments of two local health districts (LHDs) were surveyed, to determine current perceptions and understanding of the ILS in place and of departmental SC.

At the survey time, the departments had multiple reporting systems, at three levels:

- Inhouse/departmental
  ILS including: paper‐based reporting forms for actual and near‐miss incidents and also departmental non‐radiation incidents; RO morbidity and mortality QA review meetings; RT senior staff meetings discussing all incident reports; and ROMP error discussion meetings.
- Organisational
  An LHD‐level electronic platform, the Incident Information Management System (IIMS), for all actual incidents and near‐miss reports of any type.
- Mandatory bodies
  Higher mandatory reporting at specified dose deviation levels; to the hospital/department Radiation Safety Officer (RSO) for dose deviations over 5%, and/or the NSW Environment Protection Authority (EPA), for greater than 10%, as per ROPS recommendations, state wide incident management policies and EPA legislation.^7^, ^17^

Incident classification followed the ROPS recommendations.^7^ The departments had similar processes and protocols around incident reporting, monitoring, learning and QI. Each professional discipline held monthly quality/safety meetings separately, with cross‐MDT discussion and collaboration for learning being mainly at inter‐group senior (management) levels.

The aims of the survey were to identify staff understanding and use of the ILSs, any barriers to reporting and any needs for process change or departmental learning, as well as perceptions of SC. The findings prompted a QI project in one of the LHDs to evaluate and improve the ILS.

## Method

### Survey of ILS and SC

Current understanding of ILSs and attitudes towards SC were evaluated via an anonymous online survey, distributed to Radiation Oncology professionals (all ROs, RTs and ROMPs) within radiation oncology departments in the two LHDs, Western Sydney (WSLHD) and Nepean Blue Mountains (NBMLHD). The survey took less than 15 min to complete. The project received ethics approval from each LHD's human research and ethics committees.

Survey ‘gatekeepers’ were RTs who distributed the survey invitation to all RTs and ROMPs at WSLHD in October 2018 and all NBMLHD staff in December 2019. Distribution to WSLHD ROs was via RO administrative staff in February 2019. The survey was open for 2 weeks; gatekeepers sent reminder emails with current response rates on days 7 and 12. Participants were informed that completing the survey indicated consent to participate in the study.

The survey content was based on radiation oncology SC surveys from the literature.^6^, ^11^, ^13^ Eleven staff across the MDT initially piloted the survey, then it was sent out on a larger scale. The survey captured occupation, years worked and role level; the last two were largely removed from analysis to ensure anonymity.

The REDCap electronic data, capture tools hosted by The University of Sydney,^18^, ^19^ were used to collect anonymous responses and manage the study data. Responses were exported to IBM SPSS^20^ for quantitative analysis, with results compared and summarised using descriptive statistics. Open‐ended questions were evaluated to derive any key themes.

## Results

### Characteristics of respondents

Invitations were sent to 150 Radiation Oncology professional staff across WSLHD and NBMLHD; 95 (63%) responded, with similar overall response rates for each LHD (65% and 57% respectively). For the professional cohorts, response rates were 71%, 67% and 34% for RTs, ROMPs and ROs respectively.

One respondent was excluded due to partial survey completion. The distribution of the 94 participants was 73% RTs, 15% ROMPs and 12% ROs, while staffing distribution at that time was 65%, 14%, and 21% respectively.

Survey results between the two LHDs were not significantly different, utilising a Z test for two populations (*P* < 0.05), so were combined for analysis to further protect anonymity.

### Knowledge of incident reporting systems

Respondents showed various levels of ILS understanding and utilisation across the MDT. Table 1 shows the differences and similarities between the professional groups. Overall, 97% of respondents were aware of incident reporting. Those not familiar were all in their first employment year as RT Interns or RO Registrars.

**Table 1:** Understanding of incident reporting and learning system.

Survey questions	Response	RT *n* = 69 (71%)		ROMP *n* = 14 (67%)		RO *n* = 11 (34%)		Total *n* = 94 (63%)	
		*n*	%	*n*	%	*n*	%	*n*	%
How many reporting systems in use are you aware of in your department?	1	9	13%	5	36%	2	18%	16	17%
	2	37	54%	5	36%	4	36%	46	49%
	3	18	26%	2	14%	1	9%	21	22%
	4	2	3%	1	7%	0	0%	3	3%
	Don’t Know	3	4%	1	7%	4	36%	8	9%
Please state the name of the reporting system/s you know of in use in the department	No response	3	4%	0	0%	1	9%	4	4%
	Don’t know	2	3%	2	14%	2	18%	6	6%
	In‐house/Departmental‐level only (e.g.: in‐house systems/meetings)	7	10%	0	0%	0	0%	7	7%
	Organisational level only (IIMS)	20	29%	5	36%	4	36%	29	31%
	Mandatory bodies only (RSO/EPA)	0	0%	0	0%	0	0%	0	0%
	Departmental and organisational level	28	41%	6	43%	1	9%	35	37%
	Organisational level and mandatory reporting	2	3%	0	0%	2	18%	4	4%
	Departmental, organisational and mandatory reporting dose deviation level (e.g.: RSO/EPA)	7	10%	1	7%	1	9%	9	10%
Do you feel you can correctly Identify Actual Incidents vs Near Miss Incidents classification?	Yes	39	57%	10	71%	5	45%	54	57%
	No	3	4%	0	0%	3	27%	6	6%
	Some of the time	27	39%	4	29%	3	27%	34	36%
Do you feel you can correctly identify radiation incident sub classification type in relation to the nine recommended categories within the radiation oncology practice standards?	Yes	47	68%	10	71%	5	45%	62	66%
	No	4	6%	2	14%	5	45%	11	12%
	Some of the time	18	26%	2	14%	1	9%	21	22%
Are you aware of a formal investigation system of actual or near miss radiation incidents in your department such as root cause analysis?	Yes	53	77%	9	64%	2	18%	64	68%
	No	16	23%	5	36%	9	82%	30	32%

EPA, environment protection authority; IIMS, incident information management system; RO, radiation oncologist; ROMP, radiation oncology medical physicist; RSO, radiation safety officer; RT, radiation therapist.

The LHDs utilised three levels of reporting, as above: inhouse, organisational and mandatory. The largest respondent group (49%) acknowledged two systems in use. However, the professional cohorts showed differences in identifying systems. RTs (41%) and ROMPs (43%) largest responses identified dual systems at departmental and organisation levels. The RO cohort primarily stated organisational only (36%) or organisation and mandatory reporting (18%). Only 10% of respondents acknowledged all three.

All cohorts indicated high confidence levels in categorising errors once identified (Table 1), with ROMPs having the strongest confidence (71%) for both category and sub‐categorisation.

### Utilisation and barrier perception of the current system

Overall, 51% of respondents had reported an incident to one or more systems in the six months before the survey (Table 2). Of the 46 staff who had not reported, 74% stated they had not observed or identified an error. The rest had identified an error but not reported, mostly because they had escalated to more senior staff who investigated and reported, or another team member had completed the incident report. Two respondents were aware of some near‐miss incidents that were not reported, and only one respondent indicated they chose not to report.

**Table 2:** Utilisation and barriers to reporting.

Survey questions	Response	RT *n* = 69		ROMP *n* = 14		RO *n* = 11		Total *n* = 94 (63%)	
		*n*	%	*n*	%	*n*	%	*n*	%
Have you submitted an actual or near miss incident report in the last 6 months?	Yes	44	64%	3	21%	1	9%	48	51%
	No	25	36%	11	79%	10	91%	46	49%
What is the reason you did not submit a report in the last 6 months? Asked only if previous response was no	Choose not to report	0	0%	1	7%	0	0%	1	2%
	Did not notice, observe or discover any incident or near‐miss event in the past 6 months	22	32%	6	43%	6	55%	34	74%
	Informed team leader who investigated and submitted the report	2	3%	1	7%	2	18%	5	11%
	Other open answer provided	1	1%	3	21%	2	18%	6	13%
Number of barriers reported	1	19	28%	5	36%	4	36%	28	30%
	2	13	19%	2	14%	2	18%	17	18%
	3	8	12%	1	7%	4	36%	13	14%
	4	1	1%	0	0%	0	0%	1	1%
	No perceived barriers	28	41%	6	43%	1	9%	35	37%
What do you find is the biggest obstacle to you reporting actual or near miss radiation incidents in your department? (Multiple response allowed)	Takes too long	23	33%	1	7%	5	45%	29	31%
	System is hard to access	13	19%	1	7%	5	45%	19	20%
	Don’t know how to use/or understand the system	12	17%	2	14%	7	64%	21	22%
	Don’t see the benefit of reporting	4	6%	1	7%	0	0%	5	5%
	Fear of negative action towards self or others	14	20%	3	21%	0	0%	17	18%
	I do not think there are any obstacles to reporting in my department	28	41%	6	43%	1	9%	35	37%
	Other open answer provided	6	9%	4	29%	2	18%	12	13%

RO, radiation oncologist; ROMP, radiation oncology medical physicist; RT, Radiation therapist.

Overall, 37% of respondents perceived no barriers within the current system. However, 59 staff did perceive one or more barriers (Table 2). The most significant stated barrier was the time it takes (31%), then lack of knowledge/understanding of the system and its use (22%) and that it was hard to access (20%). Potential improvements to safety culture were indicated by the 18% of respondents that stated a barrier was related to fear of adverse action, and the 5% that did not see a benefit to reporting. These results show the most significant barriers were related to the system rather than departmental SC.

### Preferences for feedback and learning

Respondents ranked their preference for learning and feedback (Table 3). The preferred method overall was an all‐staff MDT meeting with either mandatory or open attendance. Next was for selected staff to attend incident meetings, that is representatives from different work areas/groups who report back to others; 54% acknowledged this as the primary method currently utilised across the departments. RT and RO cohorts had similar ranking preferences for learning options. ROMPs showed some differences, such as a higher preference for newsletters to disseminate relevant information and lower for in‐service training.

**Table 3:** Preference for feedback and learning.

Preference rankings	RT	ROMP	RO	Total
All staff attendance at MDT incident reporting meeting. Either mandatory meeting or open attendance	1st	1st	1st	1st
Selected staff attendance at incident reporting meeting (e.g.: Team leaders, or safety/quality team)	2nd	3rd	2nd	2nd
Attending in‐service training	3rd	6th	4th	3rd
Newsletter or email notification	4th	2nd	3rd	4th
Word of mouth	5th	4th	5th	5th
None at all	6th	5th	6th	6th

RO, radiation oncologist; ROMP, radiation oncology medical physicist; RT, Radiation therapist.

### Safety culture and learning capacity

The majority of staff (66%) feel encouraged to report, with 60% feeling comfortable reporting (Table 4). Most respondents (69%), but least strong for the ROMP group, thought their department practised a no‐blame culture (Table 4). Sixteen respondents stated their department did not practice a no‐blame culture, eight having either personally received, or witnessed others receive, adverse action, with two others declining to answer. Regarding assigning cause and blame between staff and processes, 37% of respondents gave this as 50%/50% staff/process, followed by 29% at 25%/75%. The majority of respondents (71%) gave positive responses to departmental learning capacity from reported incidents, with the RO cohort having the strongest perception.

**Table 4:** Safety culture and learning.

Survey questions	Response	RT *n* = 69		ROMP *n* = 14		RO *n* = 11		Total *n* = 94 (63%)	
		*n*	%	*n*	%	*n*	%	*n*	%
Please rate how encouraged you feel to report actual or near miss radiation incidents within your radiation oncology department?	Very discouraged to report	1	1%	0	0%	0	0%	1	1%
	Discouraged to report	5	7%	2	14%	0	0%	7	7%
	Neutral	14	20%	7	50%	3	27%	24	26%
	Encouraged to report	26	38%	3	21%	6	55%	35	37%
	Very encouraged to report	23	33%	2	14%	2	18%	27	29%
Do you feel comfortable reporting actual or near miss radiation incidents within your radiation oncology department?	Very uncomfortable reporting	7	10%	0	0%	0	0%	7	7%
	Uncomfortable reporting	6	9%	2	14%	1	9%	9	10%
	Neutral	13	19%	6	43%	3	27%	22	23%
	Comfortable reporting	19	28%	3	21%	6	55%	28	30%
	Very comfortable reporting	24	35%	3	21%	1	9%	28	30%
Do you feel that your radiation oncology department practices a culture of no‐blame when errors are reported?	Yes	52	75%	6	43%	7	64%	65	69%
	No	9	13%	6	43%	1	9%	16	17%
	Other open answer provided	8	12%	2	14%	3	27%	13	14%
Have you ever personally received or witnessed other staff members receiving negative action towards them due to a reported radiation incident or near miss?	Yes	14	20%	4	29%	1	9%	19	20%
	No	50	72%	9	64%	10	91%	69	73%
	Do not wish to answer	4	6%	0	0%	0	0%	4	4%
	Other open answer provided	0	0%	1	7%	0	0%	1	1%
After an actual or near miss radiation incident report is submitted, in your opinion, the cause/blame is mostly assigned to:	0% on staff member, 100% on current process	6	9%	1	7%	0	0%	7	7%
	25% on staff member, 75% on current process	18	26%	2	14%	7	64%	27	29%
	50% on staff member, 50% on current process	28	41%	5	36%	2	18%	35	37%
	75% on staff member, 25% on current process	13	19%	2	14%	0	0%	15	16%
	100% on staff member, 0% on current process	1	1%	0	0%	0	0%	1	1%
	Other open answer provided	3	4%	4	29%	2	18%	9	10%
How well do you believe your radiation oncology department is willing and able to learn from previous radiation actual and near miss incidents? Such as making positive process changes and or implementing appropriate training and education when necessary	Unable to learn from previous incidents	0	0%	1	7%	0	0%	1	1%
	Minimal ability to learn	4	6%	4	29%	0	0%	8	9%
	Neutral	16	23%	2	14%	0	0%	18	19%
	Some demonstrated ability to learn	29	42%	4	29%	5	45%	38	40%
	Demonstrated ability to learn from previous incidents	20	29%	3	21%	6	55%	29	31%

RO, radiation oncologist; ROMP, radiation oncology medical physicist; RT, Radiation therapist.

### Qualitative results

Four areas of free‐text answers were possible for questions related to (1) barriers to reporting, (2) no‐blame culture, (3) blame association to staff versus system/process and (4) open comment around the survey.

The thematic analysis highlighted four key themes: (1) SC issues, (2) blame is situational dependant, (3) QI and learning weaknesses and (4) current department‐level and hospital‐level reporting system deficits.

Respondents mentioned SC fourteen times. Overall, these supported a departmental no‐blame culture, but some responses, mainly from RTs at frontline and management levels, indicated some staff blame others and gossip. Blame assignment to staff versus process was identified as situational influenced, for example actual incident versus near miss, or perceived laziness of staff member/s involved.

Nine open responses indicated inadequacies in the current reporting system, with 11 perceiving weakness in QI and learning. The main concerns were that the more general organisational reporting systems do not fit radiation oncology needs, and reports disappear into the system with no feedback or learning. Others indicated the analysis and learning do not focus on the actual error‐causing problems and perceptions that education is often not prioritised, with minimal preventive measures to mitigate future risk.

### Quality improvement project

The survey findings were used to inform a QI project actioned in one LHD to evaluate and improve their current departmental (level i) ILS. An MDT QI project team was established to design, create and develop an electronic reporting system to suit departmental needs. This was guided by recommendations from literature, ROPS, barriers and other factors identified in the survey.

### Application of survey findings to improve local ILS

#### Electronic report development

A new customised electronic departmental‐level reporting system was locally developed on the Varian Aria^TM^ oncology information system (Varian Medical Systems, Palo Alto, CA, USA) to improve use and support enhanced analysis and department learning and feedback. Integration into Aria^TM^ was designed to reduce the following barriers: the time it takes to report, lack of understanding of system and use, and easier system access. It also enabled increased communication to appropriate staff and report extraction into Microsoft Excel^TM^ and Microsoft Power BI^TM^ (business intelligence/data analytics software) to increase data tracking and visualisation.

#### Report analysis changes

A dedicated MDT incident triage team was established and trained to support management. This provided a coordinated, centralised, structured and rapid approach to analysis and recommendations from reports.

#### Feedback and learning

Relevant meeting structures and attendance were changed, with MDT representatives in all meetings to ensure shared learning and discussion across professions. This increased communication with and between staff. Reports were easily available to all, since they were in a readily accessible electronic system. Quick feedback loops were introduced to staff involved in observed errors or barrier detection point fails and for urgent process changes. During COVID‐19 restrictions, a newsletter was used to provide regular feedback when meetings were not feasible.

#### Focussed education on how and what to report

A three‐month pilot phase tested the new system within WSLHD's smaller campus to ensure that access and use were straightforward. The system was implemented across the whole LHD. Mandatory training for all staff included how to use the new system and what to report on. A protocol non‐compliance category was introduced to report near‐miss errors which had evaded one or more barriers before being found within QA pathways before the first treatment fraction. This was to strengthen knowledge of any systematic QA weaknesses.

## Discussion

This work identified perceptions of SC and ILSs and barriers to reporting incidents in two Australian LHDs’ radiation oncology departments. The departments surveyed participated in joint tumour stream QA meetings and had similar in‐house ILSs. Most responding ROs worked across both LHD's, whereas only a few ROMPs and no RT staff did. The results show varied knowledge and understanding of the complete incident reporting systems, structure and associated learning.

By profession, the survey response rate was highest for RTs, followed by ROMPs, in both LHDs. Although errors and incidents can occur at any point in the patient pathway, detection is highest at treatment delivery and QA checkpoints, performed most frequently by RTs, followed by ROMPs.^21^ Therefore, their greater use of reporting may influence higher response rates. RO staff primarily received reports either classified as actual incidents, with incorrect radiation delivery, or when high‐risk near‐miss incidents have been reported and discussed. Overall, RT and ROMP response rates were high compared to other literature‐reported surveys of medical professionals, for example Cook et al.^20^ presented median response rates of 59% to postal surveys of healthcare professionals.^22^ Cunningham et al.^21^ found a lower response rate (35%) for physicians.

In the current work, professional cohorts showed differences in using and understanding the various ILSs (Table 1). RTs and ROMPs predominantly identified departmental and organisational systems, with ROs primarily identifying organisational and mandatory reporting. This reflected different groups main uses of systems, with RTs and ROMPs being predominantly involved in in‐house and organisational reporting and in‐house QA meetings for feedback and learning. ROs primarily used the organisational reporting systems and, when necessary, provided information for reports to mandatory bodies. In‐house system reporting capacity extends more widely and is more specific to radiation oncology needs than the organisational system. However, each serves different but overlapping purposes with other capabilities. Hence, the departments used both in parallel.

Differences in each cohort's use of reporting systems and feedback and learning loops may have influenced their different responses. ROs and ROMPs groups more frequently acknowledged mandatory reporting. These groups are heavily involved in investigations and decisions, alongside the RT management teams. When dose deviations requiring such reporting occur, ROMPs quantify dose deviations and prescribing ROs determine whether there are any clinical consequences to the patient and they also further provide decision‐making around any changes to the patient plan or symptom management and to facilitate open disclosure to the patient. Fewer of the RT cohort mentioned mandatory reporting, which occurs after the initial report and involves investigations primarily completed by ROMPs, RT management and RO collaboration.

All professional groups had limited awareness of the overall ILS processes, systems and intended purposes, indicating potential learning opportunities. Each cohort only showed a strong understanding of the system/s most utilised by their group. The differences indicated potential for improved learning across departments to give a more interdisciplinary collaborative approach to the overall ILS.

Some findings indicated potential areas for departmental education and learning around description and categorisation of errors. Increased reporting accuracy should increase data reliability and more robust trend analysis from reported incidents.^23^

The question ‘state the name of the reporting system/s you know of in use in the department?’ had few respondents (14%) stating any mandatory bodies. However, mandatory bodies were then mentioned in open answers by more staff, indicating staff have potentially interpreted the initial question as ‘within the department’, that is internal systems and not external reporting. This might have been better worded as ‘ … that are used by the department’.

Reporting to any ILS was predominantly by the RT staff followed by ROMPs, supporting the literature that frontline staff are more likely to detect incidents or errors at QA checkpoints.^21^, ^23^, ^24^ It is positive that staff are willing to report when they are aware of an error, with only one respondent stating they chose not to and two indicating that sometimes near misses were not reported.

Overall, 63% of respondents perceived one or more barriers to reporting within the current systems. The leading stated reason was the time involved, then lack of knowledge/understanding of the system or its use, or access difficulty. Obstacles related to negative SC, such as fear of negative actions or not seeing the benefit of reporting, had lower responses. It was promising that barriers relate to the structure, format and use of the current ILSs rather than SC awareness in staff or the perception of how the department treats safety issues. Ford et al.^3^ noted that electronic ILSs, customised to radiation oncology, reduce reporting barriers. ILS success is related to appropriate resources and utilisation, partnered with staff understanding and confidence that the SC is just and equitable.^25^

Respondents' perceptions of SC and learning were stronger towards positive SC than negative, for example two‐thirds felt encouraged to report and comfortable reporting. A no‐blame culture was perceived by most respondents, with 73% not having witnessed or received adverse action. Two‐thirds of respondents perceive cause and blame after an error to be attributed 25–50% to staff and 50–75% to processes. This further supports a no‐blame culture. Our findings are similar to those of Bolderston et al.^6^ Overall, the responses support positive SC and the ability to learn. However, as some respondents perceived blame culture or negative SC, there is still room for improvement.

The thematic review of the free‐text answers provided insight into some of the issues indicated in the quantitative results. There was a strong focus on developing changes to the ILS regarding feedback and learning pathways. Changes included developing an in‐house electronic reporting system that fit the scope of reporting needed within radiation oncology and provided fast consistent data analysis. This was developed and implemented in the ensuing QI project.

The newly developed electronic reporting system is integrated onto the Aria^TM^ platform, with rapid collaborative MDT triaging to manage reports in real time. This enables more effective education and reminders to staff when needed, ensuring continuous feedback between monthly meetings. Meetings are now MDT‐based rather than professionally separated and are open for anyone to attend. Hence feedback and learning pathways have a more collaborative approach with increased knowledge of all systems in each group. The meeting purpose is to review themes, discuss learning opportunities from reports and potential areas for improvement if process areas were regularly failing or of high risk and provide recommendations for working parties to consider. The overall goal was to improve feedback loops to the whole MDT, to help promote continued and increasing SC and learning and assist in QI decision‐making.

Furthermore, now that all data capture is electronic, it is more comprehensive and consistent, and visualisation is improved. This helps to provide information for department educational needs to increase learning or identify when QI projects or QA checkpoint modifications are needed.

In developing the new in‐house system, the need to reduce the three most significant perceived barriers to ILSs use was considered, the lack of knowledge/understanding of available systems, that access was difficult, and the system reporting time required. A clear structure was created to identify which system to report to, in‐house only or in‐house and hospital‐wide. The in‐house system was designed to be easy to access, quick to fill in and to readily provide all relevant patient data to reduce the number of data fields a reporter needs to complete compared to the previous paper‐based reporting forms. The incident triage team review in‐house system reports within 24 h of creation. When a report (near‐miss or actual incident) requires reporting to hospital systems, they coordinate this with all relevant information, including ROMP dosimetry reports, to assist the initial reporter. This has reduced the time involved and increased staff understanding of how to use and report into the hospital‐wide system. The in‐house system has streamlined the ROMP portion of dose difference evaluation and reporting and provided clearer and faster pathways if an incident is RSO or EPA reportable.

Thus, the survey helped to understand the departmental SC and how staff perceive and use the current ILSs, which helped guide decisions around design and changes to the ILS and the change implementation process. It has also provided support for changes to learning and educational needs required across the MDT to ensure a comprehensive, collaborative and open approach to ILSs, reduce barriers, increase reporting and increase the positive SC.

The new system has been in use across the whole WSLHD for seven months and ten months at the pilot campus. There are plans to survey again later to evaluate accumulated experience and the need for any further QI.

## Conclusion

A survey of perceptions of SC and understanding of ILSs established a baseline understanding within two LHDs. In one LHD, the results led to the development of a QI project that significantly improved the ILS. Major changes were implemented to aspects of reporting and to the feedback and learning portions of the ILS, as the survey had highlighted barriers to reporting and areas to improve feedback and learning across the department. The study findings provide a reference for future evaluations of ILS and SC that may identify continued improvements as the impact of the changes continues to be assessed, including further regular surveys, review of data accuracy in reports and trend analysis of incidents.

## Conflict of Interest

There are no conflicts of interest.

## Funding Information

No funding supported this project.

## Ethics Statement

Ethics approval WSLHD from QA/QI Committee on 15 February 2019. Approval: 1812‐02. Ethics approval NBMLHD from Appolo Committee on 15 November 2019. Approval: 19‐42(A).
